# Management of neurogenic bladder dysfunction in children update and recommendations on medical treatment

**DOI:** 10.1590/S1677-5538.IBJU.2020.0989

**Published:** 2021-02-28

**Authors:** Cristian Sager, Ubirajara Barroso, José Murillo B., Gabriela Retamal, Edurne Ormaechea

**Affiliations:** 1 National Hospital of Pediatrics Service of Urology Buenos Aires Argentina Service of Urology, National Hospital of Pediatrics Prof. Dr. P. J. Garrahan, Buenos Aires, Argentina; 2 Universidade Federal da Bahia - UFBA Departamento de Urologia Salvador BA Brasil Departamento de Urologia, Universidade Federal da Bahia - UFBA, Salvador, BA, Brasil; 3 Escola Bahiana de Medicina Salvador BA Brasil Escola Bahiana de Medicina (BAHIANA), Salvador, BA, Brasil; 4 Universidade Federal de Juiz de Fora - UFJF Juiz de Fora MG Brasil Universidade Federal de Juiz de Fora - UFJF, Juiz de Fora, MG, Brasil; 5 Hospital e Maternidade Therezinha de Jesus Faculdade de Ciências Médicas e da Saúde de Juiz de Fora (HMTJ-SUPREMA) Juiz de Fora MG Brasil Hospital e Maternidade Therezinha de Jesus da Faculdade de Ciências Médicas e da Saúde de Juiz de Fora (HMTJ-SUPREMA), Juiz de Fora, MG, Brasil; 6 Hospital Roberto del Rio Service of Urology Santiago Chile Service of Urology, Hospital Roberto del Rio, Santiago, Chile; 7 Italian Hospital Service of Urology Buenos Aires Argentina Service of Urology, Italian Hospital, Buenos Aires, Argentina

**Keywords:** Urinary Bladder, Neurogenic, therapy [Subheading], Child

## Abstract

**Introduction:**

Defective closure of the neural tube affects different systems and generates sequelae, such as neurogenic bladder (NB). Myelomeningocele (MMC) represents the most frequent and most severe cause of NB in children. Damage of the renal parenchyma in children with NB acquired in postnatal stages is preventable given adequate evaluation, follow-up and proactive management. The aim of this document is to update issues on medical management of neurogenic bladder in children.

**Materials and Methods:**

Five Pediatric Urologists joined a group of experts and reviewed all important issues on “Spina Bifida, Neurogenic Bladder in Children” and elaborated a draft of the document. All the members of the group focused on the same system of classification of the levels of evidence (GRADE system) in order to assess the literature and the recommendations. During the year 2020 the panel of experts has met virtually to review, discuss and write a consensus document.

**Results and Discussion:**

The panel addressed recommendations on up to date choice of diagnosis evaluation and therapies. Clean intermittent catheterization (CIC) should be implemented during the first days of life, and antimuscarinic drugs should be indicated upon results of urodynamic studies. When the patient becomes refractory to first-line therapy, receptor-selective pharmacotherapy is available nowadays, which leads to a reduction in reconstructive procedures, such as augmentation cystoplasty.

## INTRODUCTION

The most common etiology in neurogenic bladder (NB) in children is spina bifida (SB) and the most frequent malformation is lumbosacral myelomeningocele (MMC) (1). Patients with neurogenic bladder may present urinary incontinence, urinary tract infections (UTI), vesicoureteral reflux (VUR) and eventually, renal scarring and renal failure.

Without treatment, up to 70% of patients may develop urologic problems during the first years of life, and less than 5% become continent (2, 3). The main goals of treating the urinary tract are the preservation or improvement of renal function, and the prevention of UTI and urinary tract deterioration. Until recently, there was controversy over which approach was better for the initial management of NB: proactive or expectant approach (4, 5), however, the balance seems to be in favor of the first, since with a proactive approach there is a reduction in chronic renal disease (CRD). The most proactive interventions with the use of clean intermittent catheterization (CIC) and pharmacotherapy have become the cornerstone of early management of NB (6), leaving the most invasive procedures and reconstructive surgeries for older patients, even at ages reaching adolescence.

The aim of the present document is to update the main topics and subtopics discussed by international guidelines on the management of neurogenic bladder in children and to provide useful medical recommendations on major issues based on the best scientific evidence available. The focus will be on diagnosis, evaluation and medical treatment of children with neurogenic bladder.

## MATERIAL AND METHODS

**Figure 1:**
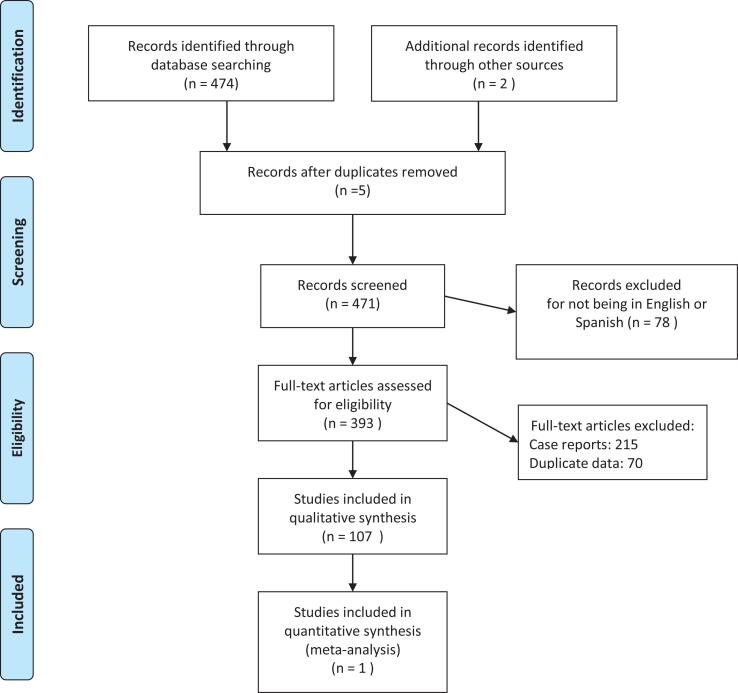
The figure shows the search strategy of the present review.

In order to adapt the guidelines analyzed for the construction of the present document, the following instruments were used “Clinical practice guidelines” (7) and “The Guideline Implementability Appraisal (GLIA)” (8). In addition, research was done following suggestions made by international referents and by the International Children's Continence Society (ICCS). Furthermore, a thorough literature search of systematic reviews on different subtopics related to neurogenic bladder in children was carried out. With the purpose of selecting the practical clinical guidelines and articles, the following inclusion criteria and parameters were used: Documents that contain in their title or abstract at least one of the following MeSH (Medical Subject Headings) terms: “Spina Bifida, Neurogenic Bladder in Children” were analyzed and selected in Medline, Pubmed and Cochrane. This search comprised articles published up to and including the year 2020. The documents that were not available in English or Spanish were excluded. The documents with a complete version that could not be recovered were also excluded. Each topic of the review was analyzed independently by each author of the present document. The search strategy is shown in Figure-1.

All the members of the primary developer group focused on the same system of classification of the levels of evidence in order to assess the literature and the recommendations following the GRADE system (modified) (9).

### Main definitions and standardizations

Neurogenic bladder is defined as any alteration of the physiologic function of the bladder due to a central or peripheral neurologic lesion. Its most common cause in children is open spinal dysraphism, such as myelomeningocele (MMC).

**Figure 2:**
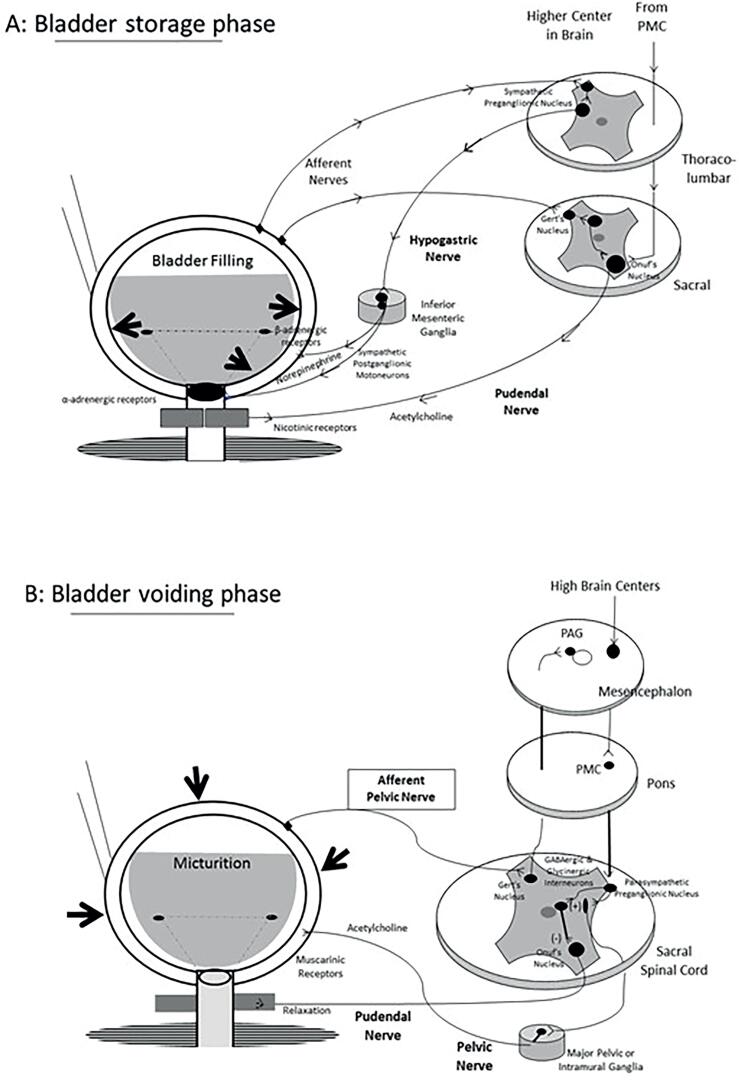
Schematic drawing of the neurourological pathway in the bladder cycle: A: Bladder storage phase and B): Bladder voiding phase.

The functions of the lower urinary tract to store and periodically eliminate urine are regulated by a complex neural control system that coordinates the activity of bladder and urethral outlet. Many neural circuits exhibit switch-like patterns of activity that turn on and off in an all-or-none manner (10). A failure of the neurulation process may induce different abnormalities called neural tube defects (Figure-2).

Symptoms in the lower urinary tract (LUTS) are considered neurogenic only in the presence of a relevant neurologic disease. According to the International Continence Society (ICS), the LUTS in neurogenic patients can be divided into three groups considering the micturition phases in which they are produced: storage, voiding, and post micturition symptoms (11). ICS as well as the International Children's Continence Society (ICCS) have published standardization documents on terminology for neurogenic LUTS (11) and non-neurogenic LUTS (12) (Table-1). Most children with NB have a reduced bladder sensation or no sensation at all, or are not aware of bladder filling or have no desire to void. Issues such as LUTS and enuresis, with different etiologies and approaches to neurogenic bladder will not be developed in the present manuscript.

**Table 1:** Standardization of Terminology for Bladder Filling and Voiding Symptoms (adapted from references 11 and 12).

Lower Urinary Tract Symptoms	
Storage Symptoms (Symptoms experienced during bladder filling)	
Symptom	Description
Urinary Frequency	Increased frequency during daytime (> 7 micturitions daily)
Nocturia	Waking up to void
Urgency	Sudden and unexpected desire to void
Incontinence	Stress: Urinary leakage on effort
	Urgency: Urinary leakage following an urgency episode
	Mixed: Urinary leakage associated with both, urgency and stress
	Continuous: Constant urinary leakage during day and night
	Enuresis: Incontinence during sleep
	Impaired Mobility Incontinence: Inability to reach toilet in time to void due to a physical of medical disability
	Impaired Cognition Incontinence: Leakage that occurs in an individual with impaired cognition without being aware of it
Bladder Sensation	Normal: Individual is aware of bladder filling
	Increased: Desire to void occurs earlier during filling phase and is more persistent
	Reduced: Desire to void occurs later despite awareness that the bladder is filling
	Absent: No sensation of bladder filling or desire to void
	Abnormal: Sensations referred as “tingling”, “burning”, or “electric shock” during filling
	Bladder Pain: Suprapubic or retropubic pain, pressure or discomfort that increases as bladder fills
**Voiding Symptoms** **(Symptoms experienced during micturition)**	
Slow Stream	Urinary stream slower than normal
Weak Stream	Urinary stream is weak
Spraying	Urinary stream passes as a spray or a split
Intermittency	Urine flow that is not continuous, stopping and starting during micturition
Hesitancy	Delay in initiating micturition
Straining to Void	Need to make intensive effort (Valsalva or suprapubic pressure) to initiate or maintain micturition
Terminal Dribble	Slow flow that trickles/dribbles at the end of micturition
Dysuria	Burning or discomfort during micturition
**Post Micturition Symptoms** **(Symptoms experienced immediately after voiding)**	
Feeling of Incomplete Emptying	Bladder does not feel empty after micturition has ended
Post micturition Dribble/Leakage	Urine loss after finishing voiding

Children with spinal dysraphism also present a neurogenic bowel, which is characterized by severe constipation, fecal incontinence, gastrointestinal motor dysfunction (13) and altered visceral sensitivity.

### Epidemiology and etiology

Congenital defects of the neural tube (CDNT) are common abnormalities that lead to a neurogenic bladder in children. Their prevalence varies in the different continents and in regions within the same continent, from 1 every 10.000 births in Alaska to 34.4 every 10.000 births in the north of China (14). In South America this prevalence is as high as 19.6 every 10.000 births. These figures increase from 20 to 50 times when there is a sibling with spinal dysraphism and 40 times if the mother suffers from it (15). A reduction in the prevalence of CDNT has been observed during the last two decades especially due to the performance of early prenatal diagnosis, the subsequent interruption of pregnancy and also the incorporation of folates in the diet (16, 17).

MMC is the most common type of CDNT and occurs with more frequency at the lumbosacral level (30-50%), followed by the lumbar and thoraco-lumbar level (20-30%, respectively), with less frequency at the cervical and thoracic level (0-5% and 5-10%, respectively) (18, 19). As to occult spinal dysraphism, its incidence is unknown, especially due to underdiagnosis; however, it is known that approximately 40% of patients will present urological symptoms at the time of diagnosis (14).

A lesion or injury in the spinal cord can also cause neurogenic bladder dysfunction in children. This etiology represents between 3 and 5% of all lesions of the spinal cord (20). Its incidence increases with age, occurring in approximately 30% of patients between 17 and 23 years of age and 53% between ages 16 and 30 (21). After the age of 3, the incidence is greater in males (4:1) (20, 21).

Of all the causes of neurogenic bladder, spinal dysraphism is responsible for up to 93% of cases (open myelodysplasia: 85%, closed/occult dysraphism: 8%), sacral agenesis, imperforate anus and lesion of the spinal cord represent 1% each, and cerebral palsy, 3%. Other less frequent causes are cerebral/spinal tumors and pelvic surgery (22).

Neurogenic bladder is present in up to 98% of children with myelomeningocele (23). The prevalence of areflexia of the detrusor muscle varies between 13 and 49.5% and hyperreflexia between 25 and 76% (24). In a group of 112 children with myelomeningocele who underwent urodynamic evaluation in 2015, Korzeniecka-Kozerska et al. found a normal function of the bladder only in 4 children (3.6%), 49.1% presented overactivity of the detrusor, 14.3% detrusor-sphincter dyssynergia, 14.3% areflexia of the bladder, and 22.3% deficient compliance of the bladder (25).

### Initial evaluation

All patients with MMC should undergo initial urologic evaluation, even those with no neuro-orthopedic alteration. The initial evaluation should provide information on the detrusor-sphincter status and define the type of dysfunction (26). 1B Strong recommendation: evidence of moderate quality. The damage of the renal parenchyma in a child with MMC, which was acquired postnatally, is preventable with adequate assessment, follow-up and proactive management (27).

Renal and urinary tract ultrasound: They should be performed as early as possible after birth. It helps detect hydronephrosis or other alterations of the upper urinary tract (26, 28).1C Strong recommendation: evidence of low or very low quality. The increased thickness of the detrusor wall does not predict urodynamic findings. Regarding unfavorable videourodynamic findings, there are not significant differences in bladder wall thickness measured at each maximum cystometric capacity, except for bladder trabeculation (29). 2B Weak recommendation: evidence of moderate quality.

Voiding cystourethrography (VCUG): It offers anatomical information on the lower urinary tract (26) and mainly on the presence or absence of VUR.

Urodynamic evaluation: It assesses the filling and voiding phases of the bladder. It is essential to carry out this evaluation since treatments depend on a specific diagnosis of the type of neurogenic dysfunction (30). 1B Strong recommendation: evidence of moderate quality. VCUG and urodynamic evaluation should be performed at least 6 weeks after closure of the defect, when it is expected that the spinal shock be overcome (31, 32). If the closure of the defect was intrauterus, it is not necessary to wait for 6 weeks. VCUG and evaluation of the upper urinary tract should be repeated at regular intervals (26). Video-urodynamics combines in only one study and instrumentation the data of the urodynamic evaluation and VCUG, with the advantage of simultaneity of the testing. It is recommended not only in the initial evaluation but also during the follow-up in children with VUR (33).

The initial evaluation is useful to identify a subgroup of patients with a greater risk of developing nephro-urologic damage (34). Creatinine estimation should be done after the seventh day of life (35). The urodynamic parameters of reduced bladder capacity and compliance and high detrusor leak point pressure (DLLP) are predictive factors of renal deterioration. In bladders with these characteristics it is less likely that VUR will be resolved (26, 36). The presence of ureterohydronephrosis and VUR is correlated with a greater risk of renal injury (31). All the patients with spina bifida should undergo Tc-99m DMSA renal scintigraphy during the first year of life, 1B Strong recommendation: evidence of moderate quality, and after 6 months of episodes of pyelonephritis. 1B Strong recommendation: evidence of moderate quality.

Patients with a neurogenic bladder require urologic control for life (26). The degree of severity in the renal damage conditions the frequency of urologic controls by imaging and urodynamic evaluation. 1B Strong recommendation: evidence of moderate quality.

This expert panel recommends: Renal and urinary tract ultrasound: every 3-4 months during the first year of life, every 6 months until age 2, and then, yearly until age 5. Urodynamic evaluation: yearly until puberty. VCUG and/or Video-urodynamic evaluation: it will be repeated yearly in the patient with VUR prior to urologic surgery, and as soon as possible in the case of patients who abandoned treatment and follow-up. For patients who are neurologic and urologically stable, with adequate bladder continence, we recommend control with renal and urinary tract ultrasound and annual evaluation of the residual urine (post CIC or voluntary micturition, as applicable) (34). There exist different situations that make it necessary to increase the frequency of urologic studies: Changes in urologic symptoms (36).

Recurrent pyelonephritis episodes. Changes in the orthopedic and neurologic signs and/or symptoms (36).

Planning of treatments to improve or achieve continence.

Neurosurgical complications (36).

Pubertal development.

In the case of patients with changes in the neurologic, orthopedic or urologic status, a neurosurgical evaluation should be carried out in order to identify: symptomatic tethered cord, syringomyelia, increased intracranial pressure caused by dysfunction of the valve system or partial herniation of the cerebral trunk and cerebellum. 1B Strong recommendation: evidence of moderate quality.

### Proactive approach/expectant approach/risk groups

It is necessary to know the factors that may predict the appearance of renal lesion in children with MMC in order to implement preventive actions. Undoubtedly, the introduction of the clean intermittent catheterization technique by Lapides et al. and the use of anticholinergic medication have made a great difference in the treatment of patients with neurogenic bladder (37). Nevertheless, when it is the best moment to apply these measures is still subject to controversy.

### Early Treatment versus Expectant Treatment

#### – Early Treatment

It consists in starting treatment before any signs of damage appear, such as UTI, hydronephrosis, renal scars, hypertension or decreased estimated glomerular filtration rate (38). This principle is based on the premise that high pressures of the detrusor muscle (>40cm H20) and pyelonephritis compromise renal function; therefore, they should be avoided right after birth (39). A comparative advantage attributed to the early start of CIC is a greater adherence and adaptation of patients and members of the family.

Anticholinergic drugs: The use of anticholinergic medication reduces intravesical pressure as well as the involuntary contractions of the bladder as shown on urodynamic studies (40). Although the use of oxybutynin has not been approved in children younger than 5 years of age, there exists enough global experience on its benefits. In a multicenter, retrospective study of children with spinal dysraphism, the mean end filling detrusor pressure was reduced from 33 to 21cm H**2**O. Over 80% of patients showed compliance greater than 70%. No serious adverse effects were reported, although constipation and facial redness were the major side effects. The oxybutynin dose indicated was: 0.2-0.6mg/kg/day given 2 or 3 times a day (41).

#### – Expectant Approach

An “expectant approach” consists in adopting a vigilant behavior before initiating any treatment. Advocates believe this approach avoids the risks of UTI generated by CIC and the side effects caused by the use of anticholinergic drugs, and it also helps reduce costs. When a treatment is implemented, renal dilatation is reversible in 92% of cases, according to Klose et al., and in 69%, as reported in a study by Kaufman et al. (42, 43). However, the latter pointed out that compliance improved only in 42% of cases and almost half of the patients with renal dilatation required bladder augmentation.

Some authors have argued that an active vigilant approach performing ultrasound and urine and creatinine tests at short intervals (every 3-6 months) may help detect changes in the upper urinary tract (UUT) early (44). These authors found deterioration of the UUT in 5% of cases. Nevertheless, as children with spina bifida require multidisciplinary assistance, it is difficult to believe that a close follow-up could be kept over time. Other authors suggest that children be evaluated taking into account the stratification of risks for UUT (45). For instance, hydronephrosis, VUR, UTI and urinary retention were considered risk factors of interest. In a study by Hopps et al., 79% of patients showed low risk at presentation, and of these, 45% became cases of high risk. Only 1.2% progressed to renal failure (45). No data about surgical and renal damage rates in the long term were informed in that report.

#### – Comparative studies

Geraniotis et al. carried out a prospective and randomized study of 21 patients with detrusor sphincter dyssynergia (46). Of the patients who kept urination (N=11), 6 presented deteriorated UUT compared with only one in the group that were treated with CIC (N=10).

Kassabian et al. retrospectively studied 26 patients with myelodysplasia treated with CIC and oxybutynin, and 56 were treated expectantly in different moments of the evaluation (47). The UUT deterioration rate was 8% and 48%, respectively.

In a retrospective analysis of 144 patients, Dik et al. observed renal scarring in 6 (48). Of these, 5 had started CIC and received anticholinergics late. Edelstein et al. performed a controlled prospective study, but unrandomized (49). A total of 35 (79.5%) out of 44 patients in the observation group progressed to UUT deterioration, whereas in the group that started treatment early, deterioration occurred in 3 out of 20. Wu et al. performed a retrospective study with a cohort of 46 children with dysraphism treated before they were one year old and 52 children after 4 years of age (50). These authors showed that hydronephrosis persistence was similar in both groups (13% and 14%, respectively). However, the rate of bladder augmentation was greater in the group of children older than 4 (11% compared to 27%, respectively).

Keye et al. retrospectively evaluated 107 patients with spina bifida. The initial treatment with CIC was performed by 39% of patients and 61% were evaluated expectantly. During the study period, 23% were changed from an expectant approach to starting CIC. The authors observed that the patients who underwent CIC were more prone to develop UTI (35.7% vs. 18.5%, P=0.045), as well as VUR (54.5% vs. 17.9%, P=0.015). As the minority group of patients underwent CIC, this was probably the group with more risk and, therefore, they developed more infection (51).

In a retrospective study, Kaefer et al. evaluated myelodysplastic patients with bladder sphincter dyssynergia and high detrusor pressure (52). They compared 18 patients treated with an early approach (between 1985 and 1990) with 27 patients treated with a conservative, expectant approach (between 1978 and 1984). They concluded that the need to perform bladder augmentation was greater in the group treated expectantly (41% vs. 17%).

In a retrospective study similar to Woo's, Delair et al. used as a result the loss of renal function >15%. A total of 252 children were assessed (53). They observed that the presence of VUR and the start of CIC after one year of age were predictors of risk.

Elzeneini et al. carried out a retrospective analysis of a group of 114 patients treated between 1997 and 2010 with an early proactive approach compared to another group of 100 treated expectantly between 1985 and 1994 (54). DMSA scans revealed renal scars in 19% and 39%, respectively.

### Risk factors

#### – Urodynamic parameters

McGuire et al. were the first to consider bladder pressure as a risk factor for renal dilatation and VUR in children with myelodysplasia (39). Of a total of 42 patients analyzed, 22 presented end-filling detrusor pressure >40cm H**2**O and 20 had pressure <40cm H_2_O. In the case of the group with lower pressure, no patient had renal dilatation and only two showed ureteral dilatation. In the group with higher pressure, 68% had vesicoureteral reflux and 81% had dilatation.

Timberlake et al. studied parameters of ultrasound (US) and video-urodynamic studies that could be associated with renal scarring on DMSA (55). Of interest, DMSA scans were performed during the first 6 months of life and renal scarring was observed in 24% of cases. The risk factors for renal scars were: trabeculation of the bladder as shown on US, end-filling detrusor pressure >40cm H_2_O, presence of residual urine >50%, pressure >10cm H_2_O at the start of bladder filling, and VUR on video-urodynamic studies.

Vesicoureteral sphincter dyssynergia (VSD) has also been identified as a risk factor of renal lesion (49, 56). In a retrospective study involving 312 patients with myelodysplasia, Ozel et al. observed that VSD and involuntary contractions were risk factors of renal scars on DMSA scans.

Other studies have not found association of urodynamic parameters and worse outcome (44, 53). Nevertheless, some aspects deserve consideration. A high pressure bladder is more associated with VUR and studies are consistent in showing that VUR is a risk factor of deterioration of UUT. Furthermore, patients with high pressure bladders began an early treatment with CIC and anticholinergic drugs, which may have favored a better outcome. Finally, a study by Dudley et al. showed that there exists a considerable variation between examiners in the performance of urodynamic studies, which may render different results (57).

#### – Ultrasonography data

The high post void residual is a relevant finding, since urine retention has been associated with UUT deterioration and it would be an indication to start CIC. However, there is no consensus over which ratio of post void residual to bladder volume would be indicative to start CIC.

Tanaka et al. measured the thickness of the bladder wall in 57 patients with MMC using ultrasonography and they compared their findings with urodynamic studies (58). Bladder wall thickness was correlated to detrusor leak point pressure >40cm H_2_O and end-filling detrusor pressure.

Sekerci et al. studied 80 children with myelodysplasia and they observed that increased bladder wall thickness correlated to urodynamic parameters, as well as to renal scarring (59). Nevertheless, these data were not confirmed in the study by Kim et al. (29). In 52 patients examined, the thickness of the bladder wall as seen on ultrasound did not correlate to video-urodynamic findings. These data were confirmed by a study performed by Muller et al. (60).

### This expert panel recommends

1. All patients should be evaluated with ultrasound after birth, (1C Strong recommendation: evidence of low or very low quality). For this test, the grade of renal dilatation and ureteral dilatation should be taken into account. The measuring of bladder wall thickness is controversial, nevertheless, this panel advises to measure it (2B Weak recommendation: evidence of moderate quality). This panel recommends that if the wall thickness is measured with a full or semi-full bladder, it may provide additional information for the interpretation of the general clinical status of the child. The cut-off value for this measure has not been established. In the presence of increased residual urine (>50% of expected capacity for age) CIC is recommended (2B Weak recommendation: evidence of moderate quality).
2. Every patient should be evaluated with urodynamic studies during the first 6 months of life (1B Strong recommendation: evidence of moderate quality). The following data should be determined: detrusor leak point pressure, end and start-filling pressure (Pdetmax), and bladder capacity.
3. Every patient should be evaluated with urinary cystourethrography and/or video-urodynamic studies (Grade B recommendation). This study helps define diagnosis and VUR classification, as well as identify signs of vesicoureteral sphincter dyssynergia.
4. Every patient with spina bifida should undergo DMSA scan during the first year of life. (1B Strong recommendation: evidence of moderate quality) and after 6 months of having episodes of pyelonephritis (1B Strong recommendation: evidence of moderate quality).
5. The early treatment with CIC helps prevent UTI, VUR and renal scarring. Anticholinergic medication will be used if urodynamic results show a need to (if there is overactivity and/or filling pressures greater than 20cm H_2_O at expected capacity for age) (1B Strong recommendation: evidence of moderate quality). Doses should be adjusted according to urodynamic results (36).
6. In children under CIC it is not advisable to indicate antibiotic prophylaxis (ATB) except during the first months of life until the parents become familiar with the CIC technique and the initial evaluation is complete. It would only be indicated in the case of patients with VUR, hydronephrosis or UTI with recurrent fever (1B Strong recommendation: evidence of moderate quality).

### Clean Intermittent Catheterization (CIC)

The introduction of CIC has helped improve the management of patients with neurogenic bladders, when Dr. Jack Lapides showed its efficacy in the long term in the 1970s (61, 62). It is a fundamental tool in the treatment of patients with alterations in bladder voiding and it contributes to avoiding renal damage and to improving urinary continence.

Indication for starting CIC: The right moment to indicate CIC is still controversial. There exist two approaches: one expectant and one proactive (63). With the expectant approach patients are periodically monitored in order to evaluate changes in UUT. CIC is indicated when there is clinical deterioration or development of hydronephrosis, upon confirmation on urodynamic studies. With the proactive management of patients, CIC is initiated even before evaluation with ultrasound and urodynamic studies is carried out, and prior to the development of changes in UUT. Those who recommend it emphasize the fact that it helps achieve better adherence, a reduction in the need of reconstruction of the urinary tract and a reduction in the risk of renal deterioration. The recommendations by ESPU and ICCS in relation to CIC are the following: The ESPU Guidelines on the management of neurogenic bladder in children and adolescents, Part I, 2019 (64), propose a proactive management with early indication of intermittent catheterization. They postulate:

- During the neonatal period, in the case of patients with spina bifida, each bladder should be considered with a potential development of overactivity and/or high filling pressure with deficient voiding, and CIC should be started soon, right after birth, since it contributes to diminishing renal complications and the need for later augmentation(64–66) (1B Strong recommendation: evidence of moderate quality).
- CIC is much better accepted by patients and parents if it is introduced early during the first days of life.
- In children with evident hypoactive sphincter without overactivity, initiation of CIC can be delayed. If CIC is delayed, then a close monitoring of UTI should be performed, as well as ultrasound of the upper tract and urodynamic studies to evaluate the lower urinary tract (1B Strong recommendation: evidence of moderate quality).
- In the case of infants with spinal dysraphism, without any signs of outlet obstruction as evidenced on urodynamic studies, CIC may be delayed, but a close follow-up should be kept.

The recommendations by ICCS for the initial diagnostic evaluation and follow-up in congenital neurogenic bladder and bowel dysfunction in children, 2012 (22), also propose a proactive management:

- Children with open spinal dysraphism that cannot empty their bladders spontaneously should perform CIC until urodynamic studies can be done safely, in general during the first 2 to 3 months of life (67–69).
- The presence of high grade VUR described in cystourethrography/video-urodynamics requires management with CIC.
- Patients with spinal cord lesion require initiation of CIC as soon as the initial clinical status is stabilized. If the patient presents spontaneous micturition, it is necessary to know at which pressure this occurs. If voiding and filling pressures of the detrusor are normal and the child empties the bladder with synergia, CIC may be discontinued in a safe way (70). If CIC is interrupted, periodical evaluations of residual urine volume should be done in order to ensure adequate bladder function. If filling and voiding pressures are high due to vesicoureteral sphincter dyssynergia, then CIC should be continued (71).

### Variables to consider in CIC

In the case of patients with spinal dysraphism it is important to indicate the use of latex-free catheters, since this pathology is highly associated with allergy to latex (72).

Cochrane Revisions and recent studies show that the incidence of UTI in patients with CIC is not affected by the use of a sterile or clean technique, coated or uncoated catheters, single (sterile) or multiple use (clean) of catheters, self- catheterization or catheterization by others, or by any other strategy (73–76).

It is described that with the use of hydrophilic catheters there is a tendency to reduce potentially pathogenic agents (bacteria) and that they have a higher level of user satisfaction (77). The investigation evidence on these issues is weak (75) and the ESPU Guidelines 2019 clarify that on the basis of current data, no statement can be made regarding which type of catheter, technique or strategy is better than others (64).

For people using CIC, the selection of the catheter will depend on personal preference, cost, portability and how easy it is to use (75).

In relation to the correction of in utero neural defect, there are no significant differences between prenatal versus postnatal closure on the need for CIC, the urinary incontinence rate, the need for reconstructive urologic surgery, or urodynamic parameters (78, 79).

### Training in CIC

The person in charge of training parents or patients of an older age to use CIC should be a trained nurse. In a neonatal unit, the newborn will not be discharged until parents can complete their training in CIC. The clean technique includes hand washing with water and soap (it does not require gloves or antiseptic products), and it should be done 5 to 7 times a day.

### Age-related selection of the catheter diameter

There exist different diameters and the choice of catheter is related to the caliber of the urethra by age of the patient: between 0 and 2 years of age, a 6 French (Fr) catheter is recommended, between 2 and 5, catheter 6 or 8 Fr, between 5-10 years old, 8-10 Fr, between 10 and 16 years old, 10 to 12 Fr, and for patients older than 16, catheters 12 to 16 Fr (80). Regardless of this description, the final selection is up to each individual.

### Types of catheters

Although it has already been said that the ESPU Guidelines 2019 state that no final statement can be made in relation to the best type of catheter to be used (64), it is important to know which types there exist:

- Uncoated catheters, made of polyvinyl (PVC) or silicone, they require lubrication for its use.
- Coated catheters with hydrophilic coatings. When these catheters are exposed to water, the outer layer attracts the water to the surface of the catheters ensuring lubrication of the urethra. These catheters are useful to minimize discomfort, in the presence of urethral stenosis or catheterizable channel stomas.
- Closed-catheters systems with pre-lubricated products that are packaged as a sterile kit containing all the equipment required to do aseptic catheterization.

There is an alternative catheter design to consider that can be useful: a catheter with a curved tip or Coudé tip to ease passage through a high neck bladder.

In addition, it is worth mentioning the Fuji catheter, made of silicone rubber, which preserves its characteristics for a long time (12 to 14 months). It is kept in a container with an antiseptic solution and a lubricant, which makes its preservation and transportation easier. It is an ideal alternative for school children performing self-catheterization.

### Self-catheterization

This is the technique by which patients perform their own bladder emptying. The patient should be mature and responsible enough to perform a successful treatment and they should have motor skills at least in one arm so that they can reach the urethra or stoma. An expert nurse should train these patients on CIC. The male patient may require time to know the correct positioning of the urethra during insertion of the catheter and the female patient needs time to locate the urethral meatus (they may use a mirror at first or digital self-examination). For the patient, this is a great step towards independence.

### Catheterizable channels

Surgically created channels can be used in the case of patients requiring CIC but whose urethra is compromised or inaccessible. They can be indicated for several reasons (81):

- Difficult access to the perineum due to physical limitations or the use of a wheelchair;
- Presence of urethral sensitivity;
- Surgery of bladder neck;
- Urethral pathology that makes access to bladder difficult (urethra atresia, urethra stenosis).

The creation of catheterizable channels can improve quality of family life, since with this option the time allotted to positioning the patient and cleaning the insertion site of the catheter is reduced (82). Also, training in the use of these channels is usually easier compared to training in CIC.

Among the channel techniques available we can mention Mitrofanoff appendicovesicostomy (which uses cecal appendix), Yang-Monti tunneled channel (using reconfigured ileum), and spiral Monti, which creates a longer tube that can be very useful in the case of obese patients (83). The ureter could also be used if the ipsilateral renal unit is excluded. The complications of these reconstructive techniques include incontinence, difficulty in catheterizing the channel and stenosis of the stoma (5 to 57%) (84).

### This expert panel recommends

1. CIC should be initiated soon, right after birth, since it contributes to diminishing renal complications and the need for subsequent bladder augmentation (35, 64, 65) (1B Strong recommendation: evidence of moderate quality.)
2. CIC should be done 5 to 7 times a day, every day after birth. Even before performing the first urodynamic or video-urodynamic studies. (1B Strong recommendation: evidence of moderate quality (36)).
3. In the case of those children with evident hypoactive sphincter with no overactivity, the initiation of CIC can be delayed. If CIC is delayed, a close monitoring of UTI should be performed, as well as ultrasound of the upper tract and urodynamic studies to evaluate the lower urinary tract. (1B Strong recommendation: evidence of moderate quality).
4. The training program in CIC includes the necessary sessions for two adults responsible for the care of the child. Training starts in the intensive care unit and continues in the Urology unit (with necessary coordination between nurses of both sectors).
5. Self-catheterization should be encouraged upon evaluation of level of development and maturity of each child.
6. The CIC diary constitutes one way of controlling adherence to CIC, and it should be monitored in every visit to the Urology unit or the Spina Bifida Multidisciplinary group.
7. If possible, hydrophilic coated catheters should be used in the context of a painful procedure, in the presence of urethral stenosis or in cases of stomas with catheterizable channels, such as Mitrofanoff.

### Second-line pharmacological therapies

When first indications in the proactive management, i.e. CIC and anticholinergic medication, do not achieve optimal therapeutic effects and there are adverse effects, the patient becomes refractory or intolerant to medication. This means there is a need to proceed to second-line therapies.

A patient is considered refractory if they meet the following criteria: (1) previous medical therapies could not help improve urine incontinence, (2) there is absence of correctible neurological anomalies evaluated on nuclear magnetic resonance (NMR), and (3) incomplete or non-satisfactory urodynamic response is shown despite the use of optimized doses (without side effects) of anticholinergic drugs at the most tolerated highest dose.

Once a patient becomes refractory, it is necessary to reevaluate the neurosurgical aspect in order to rule out any active process in the central nervous system that may be blocking the function of target organs: bladder, bowel and lower limbs. One of the most frequent entities of closed dysraphism is lipoma and the possibility of having a tethered spinal cord. Although there exist controversy over the indication of surgery in the case of complex lipomas, Pang recommends total resection for most complex lipomas with or without symptoms. With respect to asymptomatic chaotic lipomas, prophylactic resection is not currently endorsed (85).

### Scheme of double oral anticholinergic therapy

The combination of two or more anticholinergic drugs is a conservative option, although adverse effects may be potentially additive. However, this alternative is worth considering, especially in a pediatric population, in which it is convenient to postpone more aggressive measures.

In other words, in the case of patients with persistent incontinence and incomplete urodynamic response to single-agent anticholinergic therapy (oxybutynin 0.3mg/k/day or 15mg oxybutynin extended release (ER)), a second drug can be added with dose escalation, for instance Tolterodine, doses: 4mg, 2mg every 12 hours or 0.25 - 1mg twice a day. The dose should be increased until the symptoms disappear, intolerable side effects appear or until the maximum dose is reached (30mg of oxybutynin ER, 4mg of tolterodine ER). If the minimum daily dose of 10mg oxybutynin ER is not well tolerated, it is replaced by 4mg tolterodine ER.

In case there is a suboptimal response with the second anticholinergic drug (tolterodine 4mg ER) or the medication is not well tolerated, solifenacin 5 to 10mg can be used. In a study of patients with neurogenic and non-neurogenic bladder dysfunction with overactivity, Nadeau et al. reported that mean micturition volumes, cystometric bladder capacity and maximum pressure of detrusor contractions improved significantly with treatment with solifenacin. The number of incontinence episodes per day diminished significantly from the beginning of therapy until the end of the study (86).

After the third month of each change of medication or modification in the dosage, a clinical and urodynamic reevaluation is required to have an objective assessment. In addition, blood samples and electrocardiograms should be obtained at the start of treatment and every 6 months in order to detect potential toxicity with solifenacin.

Even though the use of imipramine has been reported with promising results in cases of patients refractory to anticholinergics with bladder dysfunction of different etiologies, up to date there is no solid evidence to recommend its use in pediatric populations. Furthermore, possible cardiac side effects have been reported and this should not be disregarded (87).

### ß3 adrenoceptor agonists

ß3 agonists, such as Mirabegron, are agents with a different mechanism of action, since they act on the ß3 receptors of the bladder producing relaxation of the detrusor muscle and interacting with receptors of other organs, such as the heart (88). Studies in phase 2 and 3 have reported that mirabegron significantly improves clinical symptoms of bladder overactivity in adults, which has led to its worldwide approval for this entity.

Blais et al. carried out a prospective study where they analyzed the clinical response to mirabegron for the first time in a pediatric population but with overactivity in non-neurogenic bladder. They included 58 patients refractory to behavioral therapy or urotherapy and anticholinergic medication. They showed improvement in the voided volume, urinary incontinence and a reduction in the side effect rates (89).

In 2018, JS Park et al. published their experience with mirabegron in the treatment of neurogenic bladder in children. They recruited 66 patients with NB as sequela of spina bifida who were refractory to anticholinergics. They excluded patients who had received botulinum toxin-A injection previously and they indicated mirabegron 50mg/day. The results showed a significant increase in compliance and bladder capacity. Furthermore, 37.1% of patients became completely dry after taking mirabegron and 9.1% reported adverse effects (90).

Krhut et al. evaluated efficacy and safety of mirabegron 50mg/day in the treatment of neurogenic overactivity of the detrusor in patients between 18 and 65 years old with neurogenic bladder as sequela of spinal cord injury or multiple sclerosis. They concluded that in the group of patients treated with mirabegron there was a significant increase in the volume during the first contraction, an improvement in compliance, a non-significant increase in capacity and a tendency to a reduction in urine leaks (91).

In children with neurogenic bladder refractory to first and second-line therapies, mirabegron, as an adjuvant, increases bladder capacity, reduces intravesical pressure and achieves continence in a great number of patients. It is a well-tolerated drug, with no adverse effects, which implies reducing or postponing the need for bladder augmentation (92).

### The role of α-blockers

Reports have shown that agents such as doxazosin, tamsulosin and alfuzosin do not modify significantly DLPP in children with neurogenic bladder (Momper, 2014). Further studies should focus on tests with a range of doses using objectively measured, clinically meaningful endpoints.

### Botulinum Toxin Type A

In children/adolescents with neurogenic bladder refractory to first-line therapy, injections with botulinum toxin-A (BTX) in the detrusor muscle should be considered in order to have a safe bladder with adequate capacity at low pressure.

Hascoet et al. performed a systematic review in children with neurogenic bladder and showed a wide range of results: continence could be achieved in 32% to 100%, maximum detrusor pressure decreased between 32% and 54%, maximum cystometric capacity increased between 27% and 162%, and compliance improved between 28% and 176%. OnabotulinumtoxinA (maximum doses 300 IU injected into 20-30 different bladder sites) seemed to be more effective mainly in overactive cases, compared to hypertonic patterns (93).

Some authors have reported that injections with botulinum toxin-A in patients with neurogenic bladder due to MMC can be ineffective if the detrusor muscle is fibrotic, of low compliance and with loss of contractility (94). Nevertheless, we should not underestimate these findings, since some authors state that as there is increase in capacity, there is also improvement in compliance. It has been demonstrated that injections with BTX into the trigone seem to be safe regarding VUR and the changes in the upper urinary tract (95).

Among the main advantages of injections with botulinum toxin-A we may mention: it is a minimally invasive method, it requires sedation and a short hospital stay, it has very low adverse effect rates: mild hematuria and very few cases of UTI reported (more related to cystoscospy). The main disadvantage is its transient effect, with no more than 6 months of therapeutic effect, which requires re-injections, and this is a problematic issue when patients are in transition towards adult centers (96) (2A Weak recommendation: evidence of high quality).

Injections of botulinum toxin-A into the detrusor muscle in children who are refractory to antimuscarinic drugs have shown beneficial effects in clinical and urodynamic variables. We evaluated 26 children with neurogenic bladder refractory to conventional treatment. After treatment with repeated injections of intradetrusor onabotulinumtoxinA, urinary continence was achieved in up to 77%, the mean bladder capacity and the mean maximum cystometric capacity increased. The mean detrusor pressure at the end of filling decreased, but only compliance after the first injection improved significantly. Detrusor overactivity was attenuated, but did not disappear completely (97).

In the presence of patients refractory to antimuscarinic drugs, taking into account dosage of each drug depending on body weight, and the presentation of adverse effects, this panel of experts recommends the following possible plan:

1. Oxybutynin 0.2-0.6mg/k/day every 8 hours or oxybutynin ER+Tolterodine (0.25-1mg every 12h) or Tolterodine ER 4mg.
2. Oxybutynin 0.2-0.6mg/k/day every 8 hours or oxybutynin ER+Solifenacin 5 to 10mg a day (for adolescents).
3. Tolterodine 0.25-1mg every 12 hours or Tolterodine ER 4 mg+Darifenacin 7.5, 15mg a day (for adolescents).
4. Injections of botulinum toxin-A 6-10 IU/k: if there is overactivity or hyperreflexia (2A Weak recommendation: evidence of high quality). With or without plan 1, 2 and 3 as adjuvants. If after the first injection there are significant urodynamic and clinical changes, repeat injection nine months after the first injection. If there are no significant changes, the following option should be considered:
5. Mirabegron 25 to 50mg a day, with or without an agent from plan 1, 2 and 3 as adjuvants. Example: Tolterodine 4mg+Mirabegron 50mg day.
6. If after a clinical (CIC diary) and urodynamic evaluation at least 3 months later with one of the previously mentioned options the patient remains refractory or resistant to medication, bladder augmentation should be considered.
7. The use of α-blockers to diminish pressure in the exit tract and facilitate bladder emptying has not been tested in controlled studies in children (36) (2C Weak recommendation: evidence of low or very low quality).

The use of bowel segments in bladder augmentation has promoted important advances in finding new ways to deal with patients with non-compliant bladders. Nevertheless, there is concern regarding specific characteristics of the intestinal epithelium that can result in complications. Non-secretory bladder enlargement is considered as one of these alternatives, as it does not present the characteristic disadvantages of the secretory and absorptive function of the intestinal mucosa (98).

### The spina bifida multidisciplinary group

This group of experts considers it is necessary to count on a multidisciplinary team for a better management of patients with neurogenic bladder dysfunction.

Every health care center will select the participants in a multidisciplinary team that must very well know the commitment and responsibility that the management of these patients requires. The specialties involved in such a team are: neurosurgery, neurology, urology, nephrology, pediatrics, gastroenterology, physiatry, orthopedics, among others. In addition, we must mention nurses, physical therapists, occupational therapists, psychologists, nutritionists and social workers.

The so-called “Life Course Model” (99) describes the roles and milestones in the life of an individual during childhood, school life, adolescence and adulthood focusing on medical assistance with a multidisciplinary approach. At each stage in life there are objectives set and the health care providers can monitor or intervene in a coordinated way in view of the goals to be achieved at each step.

Multidisciplinary groups of neurogenic bladder carry out different programs to manage particular situations in several subgroups. One of these subgroups with direct influence on neurogenic bladder is the neurogenic bowel subgroup. Its treatment is fundamental for an adequate management of neurogenic bladder, especially at an early age, in order to avoid constipation and incontinence (64).

This panel of experts recommends: To initiate an early adequate program of bowel management (1B Strong recommendation: evidence of moderate quality).

The nurse in the urology unit is usually the person who gets to know the socio-cultural background of the patient better and they can detect problems in adherence, thus they can activate support networks to deal with issues such as overweight (100), irregular behavior and bowel movement habits. Furthermore, they can counsel the family and help them understand issues related to the rights of patients (101).

The dynamics of the multidisciplinary team will depend on the policy of each health care center, the availability of specialists in shared spaces and schedules, and, above all, the management of the patient by the coordinated circuit of sectors. It is of vital importance to count on a leader of the team, in general a pediatrician, who is in charge of encouraging necessary changes and adjustments among specialties and sets priorities. Furthermore, it is necessary to address the following issues: to have access to a physician of reference in inland regions, and to count on counseling for the disability certificate and accessible integrated electronic registry.

### Renal function in pediatric neurogenic bladder

The management of children with neurogenic bladder should be focused on the preservation of the renal function with the prevention of renal scars, avoiding progression to chronic renal disease (CRD). An early start of therapy leads to improve the preservation of the renal function in children with neurogenic bladder (102). Approximately 10-30% of children are born with evidence of renal disease and this figure increases considerably over time, with some even reaching renal compromise in about 50% of cases, as some reports describe (102). Therefore, accurate measures of kidney function are essential to avoid glomerular and tubular compromise.

It is recommended that the glomerular filtrate rate (GFR) be estimated together with the serum creatinine value using the Chronic Kidney Disease Epidemiology Collaboration (CKD-EPI) creatinine-based formula and the updated Schwartz “bedside” formula (CKiD 2009) for children (103, 104). Measuring the height in a patient with spina bifida is a difficult task, since many patients cannot walk, but the estimation can be done via measurement of the arms.

In children, protein excretion of <100mg/m2/day or <4mg/m2 /hour in a 24hr urine collection is considered normal. It is important to investigate proteinuria (up to 5mg/kg/day in neurogenic bladder) as a marker of renal lesion, as well as the protein/creatinine index (up to 0.2 in NB), the albumin/creatinine ratio (up to 30mg in NB) and 24hr microalbuminuria (up to 30mg/day in NB).

DMSA, 99m Technetium Dimercapto-Succinic Acid renal scans, are ideal to confirm renal scars in children with spina bifida. A history of VUR and UTI is associated to abnormal findings on DMSA in follow-ups of patients older than 10 years of age with spina bifida (105). Kanaheswari et al. identified 45 children with spina bifida who received multidisciplinary assistance during at least 2 years. Of these, 78% showed evidence of having neurogenic bladder and 35.5% developed renal failure with scarring. Most of the children were studied during the neonatal period, but 35.6% showed remission after 6 months of age (106). A total of 36% of patients with MMC under follow-up for 5 years presented renal impairment (mainly renal disease grade I) with a proactive management. Of the ones with late initiation of treatment, 42% developed renal disease (Sager et al., 2020, data not published). Despite advances in the understanding of Renin-Angiotensin-Aldosterone System (RAAS) as a participant in the mechanism of renal injury, the analysis of its serum (ACE and ACE 2) and urinary (urinary ACE) markers was not significant in patients with MMC with renal injury previously detected by renal DMSA scintigraphy (107).

### Criteria for the definition of chronic renal disease in children

- Renal damage of more than 3 months of duration defined by structural or functional alterations determined by imaging, laboratory analyses (blood or urine tests), or renal biopsy, with or without decrease in glomerular filtrate rates.
- Glomerular filtrate rate <90mL/m/1.73m²≥3 months with or without signs of renal damage previously mentioned or using the updated Schwartz formula (108).

### This panel of experts recommends

1. All patients with spina bifida should undergo renal DMSA scintigraphy during the first year of life and after 6 months of presenting pyelonephritis episodes (1B Strong recommendation: evidence of moderate quality).
2. Evaluation of renal function with estimated clearance of creatinine (Schwartz formula) or clearance measured in 24-hour urine collection. It is recommended for those patients with renal lesions as shown on DMSA and/or with proteinuria.
3. Detection of 24 hr proteinuria (>5mg/Kg/day: abnormal) and 24 hr microalbuminuria (>30mg/day). Recommended for those patients with renal lesions as seen on DMSA and/or with proteinuria.

## CONCLUSION

The aim of this document is to offer an update on neurogenic bladder dysfunction in children and recommendations on the major topics related to the management of patients suffering from it.

Neurogenic bladder dysfunction represents one of the main sequelae of defective closure of the neural tube in children. The prevalence of spina bifida is variable among regions and its most frequent type is myelomeningocele, which produces severe sequelae for life. Multidisciplinary groups of neurogenic bladder carry out different programs to manage particular conditions, such as the neurogenic bowel subgroup.

One of the most affected systems is the urinary tract, with the potential compromise of the renal parenchyma, which should be most cared for. It is necessary to assess the anatomy and function of the urinary tract in order to stratify the risk of exposure of the renal mass to adverse bladder conditions. It has been demonstrated that clean intermittent catheterization and pharmacotherapy should be implemented as early as possible in order to avoid deterioration or damage.

If patients become refractory to first-line medication, there are more selective second-line therapies, such as beta agonists and botulinum toxin-A that can delay cystoplasty, improve urinary incontinence scores and maintain safe intravesical pressures. This contributes to reducing the morbidity of reconstructive procedures and to enhancing quality of life and social inclusion.
